# Feasibility of ultra-high-dimensional flow imaging for rapid pediatric cardiopulmonary MRI

**DOI:** 10.1186/1532-429X-18-S1-P217

**Published:** 2016-01-27

**Authors:** Joseph Y Cheng, Tao Zhang, John M Pauly, Shreyas Vasanawala

**Affiliations:** 1 grid.168010.e0000000419368956Radiology, Stanford University, Stanford, CA USA; 2 grid.168010.e0000000419368956Electrical Engineering, Stanford University, Stanford, CA USA

## Background

A comprehensive congenital cardiac MRI exam with contrast enhancement typically consists of multiple scans to evaluate dynamic contrast enhancement (DCE) characteristics, physiological function, blood flow, and anatomical assessment. However, multiple sequences require skilled operators, prolong the exam, and lengthen anesthesia duration for uncooperative patients. Thus, we propose a single comprehensive *5D flow* volumetric acquisition for a simpler and shorter cardiac exam. This technique resolves the cardiac cycle (1D), contrast dynamics (1D), and flow velocities for 3D volumes. The feasibility of the proposed 5D flow method is demonstrated for cardiopulmonary imaging.

## Methods

Velocity-sensitization in 3D is integrated into a temporally resolved DCE sequence (T1-weighted RF-spoiled GRE) using 4 velocity-encoding echoes. The gradients for velocity encoding are also used to acquire intrinsic Butterfly navigators to monitor patient motion (JY Cheng et al ISMRM 2015, p451). During the acquisition with EKG monitoring, gadolinium contrast is intravenously administered. To enable compressed sensing, a pseudo-random k-t view- ordering scheme is used to acquire the k-space data (JY Cheng et al JMRI 2015). Different sampling masks are applied during each velocity-encoding echo to achieve higher temporal resolution.

The data are binned into different volumes based on the cardiac phase, contrast phase, and velocity-encoding echo. This results in a 5D flow dataset. Though highly undersampled, the dataset can be reconstructed by exploiting data redundancies in multiple dimensions (M Lustig et al MRM 2007) and by applying parallel imaging (M Uecker et al MRM 2014). With the Butterfly motion estimates, soft-gating is used for suppressing motion artifacts (JY Cheng et al ISMRM 2015, p451). Spatial wavelet and total variation for the cardiac-cycle and contrast-phase dimensions are used for compressed-sensing regularization constraints (L Feng et al MRM 2013).

Two different reconstructions are performed to highlight different features: R1) DCE dynamics and R2) flow dynamics. For R1, fewer cardiac phases and more contrast phases are reconstructed to emphasize the DCE dynamics. For R2, more cardiac phases and a single contrast phase are reconstructed to characterize cardiac function and blood flow velocities.

## Results

Angiography is depicted using R1 with a 2.1-s temporal resolution. By subtracting the baseline pre-contrast phases, the pulmonary perfusion dynamics can be characterized. The pulmonary vessels were first enhanced at 4.1 s and then at 6.1 s post-injection. Lastly, in R2, pulmonary and aortic flow can be quantified and visualized with velocity vector renderings.

## Conclusions

The feasibility of a 5D flow technique has been demonstrated to characterize contrast-enhancement dynamics and blood flow. The integrated sequence has potential to simplify the acquisition process, enable advanced high-dimensional reconstructions, and improve post-processing analysis.

## Funding

NIH R01-EB009690, NIH P41-EB015891, NIH R01-EB019241, and GE Healthcare.

**Figure 1:**
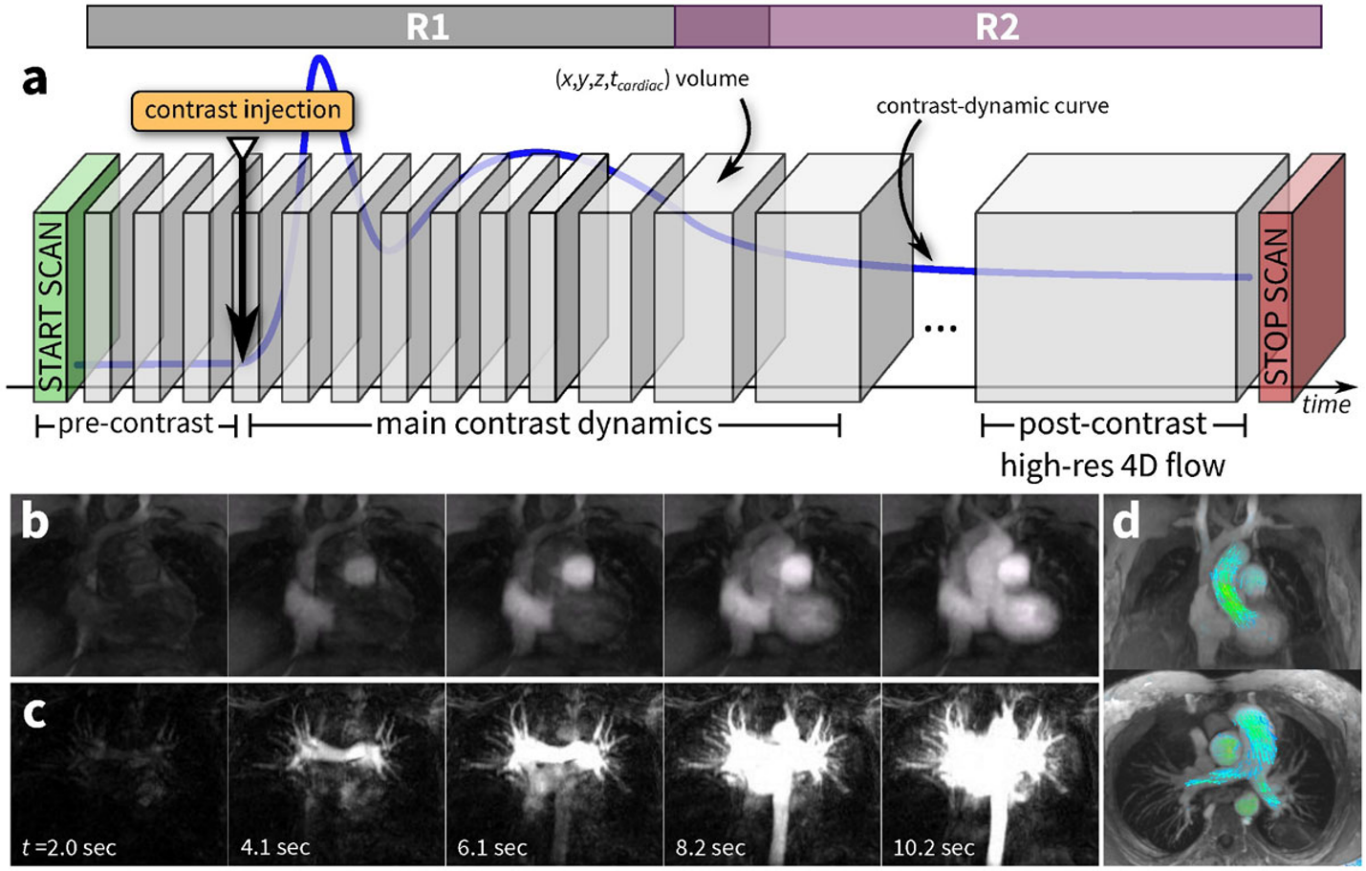
**a: method overview, b-d: results from a 24-yr-old female patient**. The 5D flow consists of a single scan. During the scan, contrast is administered (contrast curve in blue). The fast hemodynamics is captured with a high temporal resolution (in reconstruction R1). A post-contrast high-resolution dataset near the end of the scan can be generated (equivalent to conventional 4D flow imaging in reconstruction R2). Each block consists of 3D space with cardiac phases and flow quantification. In **b**, the angiography highlights cardiac function. For **c**, the reconstruction in **b** is displayed with the baseline temporal phase subtracted from each phase; this highlights pulmonary perfusion. For **d**, the cardiac-resolved volumetric imaging with blood flow information is depicted with velocity vector rendering showing aortic flow (top) and pulmonary flow (bottom).

